# EXPERIENCES WITH PARATRANSIT USE AMONG OLDER ADULTS LIVING WITH DISABILITY

**DOI:** 10.1093/geroni/igae098.4297

**Published:** 2024-12-31

**Authors:** Morgan Fique, Lindsey Mathis, Andrea Melgar Castilo, Erica Twardzik, Jason Falvey, Jasmine Cooper-Williams

**Affiliations:** University of Maryland School of Medicine, Baltimore, Maryland, United States; University of Maryland Baltimore, Baltimore, Maryland, United States; University of Maryland Baltimore, Baltimore, Maryland, United States; University of Michigan, Ann Arbor, Michigan, United States; University of Maryland School of Medicine, Baltimore, Maryland, United States; University of Maryland School of Pharmacy, Baltimore, Maryland, United States

## Abstract

Public transportation agencies are required to offer paratransit services to individuals near fixed routes, but structural barriers to use (e.g., delays in rides) may disproportionately impact older adults. This is important, as nearly 10% of urban-dwelling older adults use public transportation, many to get medical care. However, how older users interact with, benefit from, or experience barriers related to paratransit services is unclear. We completed 12 semi-structured interviews with paratransit users aged 55 and older in the Baltimore area and analyzed the transcripts using inductive thematic analysis. Two independent researchers coded each transcript. Six main themes emerged: 1) Paratransit services help older adults maintain independence and reduce social isolation by improving access to healthcare and social engagement; 2) Ableist interactions with paratransit staff and access to working equipment (e.g., wheelchair lifts) were negative determinants of paratransit use experiences; 3) Inefficiency of the service, eligibility process, and recertification process place high burdens on older riders; 4) Paratransit frequently fails to meet needs of older disabled riders who experience cognitive impairment, sensory impairment, and/or mobility device use; 5) Built and social environment factors such as sidewalk quality, weather, and crime exposure increase the burden on older riders; and 6) Formal and informal social supports are critical for older adult paratransit use. The themes identified revealed critical gaps in current paratransit systems. These findings are crucial for guiding efforts to reshape policies and practices. By addressing these barriers, we can work towards improving service inefficiencies, eliminating ableism, and better supporting older disabled riders.

